# Supplementary material for: Childhood growth and development and DNA methylation age in mid-life
Source: Clin Epigenetics. 2021 Aug 9;13:155. doi: 10.1186/s13148-021-01138-x (PMC8351141; doi:10.1186/s13148-021-01138-x)
Supplement: Supplementary file 1 — Additional file 1. Table S1. Comparison of NSHD participants included in this analytical sample versus all other participants. Table S2. Pairwise correlations between the four DNAm age acceleration markers (n = 1376). Table S3. DNAm Age Acceleration at 53 years and conditional growth based on LMS method. Table S4. DNAm Age Acceleration at 53 years and SITAR height-tempo. Table S5. DNAm Age Acceleration at 53 years and conditional growth adjusted for estimated cell composition. Table S6. DNAm Age Acceleration at 53 years and pubertal timing adjusted for estimated cell composition. Table S7. DNAm Age Acceleration at 60–64 years and conditional growth. Table S8. DNAm Age Acceleration at 60–64 years and pubertal timing. [file 13148_2021_1138_MOESM1_ESM.docx]

Supplementary Material

Childhood growth and development and DNA methylation age in mid-life

Jane Maddock PhD, Juan Castillo-Fernandez PhD, Andrew Wong PhD, George B Ploubidis PhD, Diana Kuh PhD, Jordana T Bell DPhil, Rebecca Hardy PhD

| **Supplementary table 1**. Comparison of NSHD participants included in this analytical sample versus all other participants | | | | | | | | |
| --- | --- | --- | --- | --- | --- | --- | --- | --- |
|  | Not included (n=3,986) | | |  | Included (n=1,376) | | |  |
|  | **N** | **Mean** | **Std. Dev.** |  | **N** | **Mean** | **Std. Dev.** | **p-value from t-test** |
| *Weight (kg)* |  |  |  |  |  |  |  |  |
| Birthweight | 3952 | 3.37 | 0.55 |  | 1375 | 3.40 | 0.51 | 0.11 |
| Weight at 2y | 2913 | 12.94 | 1.52 |  | 1203 | 12.88 | 1.49 | 0.25 |
| Weight at 4y | 3091 | 17.28 | 2.21 |  | 1298 | 17.12 | 2.08 | 0.03 |
| Weight at 7y | 2701 | 22.86 | 3.21 |  | 1299 | 22.70 | 2.99 | 0.12 |
| Weight at 15y | 2385 | 51.87 | 9.23 |  | 1232 | 51.68 | 9.18 | 0.56 |
|  |  |  |  |  |  |  |  |  |
| *Height (cm)* |  |  |  |  |  |  |  |  |
| Height at 2y | 2836 | 85.27 | 5.16 |  | 1174 | 85.45 | 4.81 | 0.30 |
| Height at 4y | 3020 | 103.18 | 5.21 |  | 1264 | 102.99 | 5.02 | 0.27 |
| Height at 7y | 2806 | 119.97 | 5.66 |  | 1334 | 119.95 | 5.64 | 0.92 |
| Height at 15y | 2405 | 160.29 | 7.96 |  | 1234 | 160.39 | 8.00 | 0.71 |
|  |  |  |  |  |  |  |  |  |
| *Age at menarche (y)* | 1107 | 12.99 | 1.15 |  | 617 | 13.12 | 1.26 | 0.06 |
|  |  |  |  |  |  |  |  |  |
| Body Mass index at 53 y | 1,578 | 27.49 | 5.02 |  | 1,370 | 27.35 | 4.54 | 0.41 |
| Forced expiratory volume - 1 second at 53y | 1,533 | 2.80 | 0.71 |  | 1,326 | 2.76 | 0.71 | 0.17 |
| Grip strength at 53y | 1,527 | 37.55 | 13.89 |  | 1,323 | 37.57 | 14.75 | 0.94 |
| Systolic blood pressure at 53y | 1,576 | 135.97 | 20.38 |  | 1,354 | 136.17 | 19.74 | 0.79 |
|  |  |  |  |  |  |  |  |  |
|  | **N** | **%** |  |  | **N** | **%** |  | **p-value from chi-square** |
| *Pubertal Stage at 14-15 years (men)* | | |  |  |  |  |  |  |
| Fully mature* | 309 | 23.29 |  |  | 172 | 26.22 |  | 0.15 |
| Later puberty | 1018 | 76.71 |  |  | 484 | 73.78 |  |  |
|  |  |  |  |  |  |  |  |  |
| *Socioeconomic position* | |  |  |  |  |  |  | 0.51 |
| Non-manual | 1,284 | 65.68 |  |  | 891 | 66.79 |  |  |
| Manual | 671 | 34.32 |  |  | 443 | 33.21 |  |  |
|  |  |  |  |  |  |  |  |  |
| *Smoking status* |  |  |  |  |  |  |  | 0.24 |
| Current smoker | 362 | 22.44 |  |  | 333 | 24.22 |  |  |
| Ex-smoker | 788 | 48.85 |  |  | 630 | 45.82 |  |  |
| Never smoker | 463 | 28.70 |  |  | 412 | 29.96 |  |  |
|  |  |  |  |  |  |  |  |  |
| *Physical activity in the last 4 weeks at age 53* | | | |  |  |  |  |  |
| None | 807 | 50.06 |  |  | 670 | 48.76 |  | 0.36 |
| 1-4 times | 265 | 16.44 |  |  | 253 | 18.41 |  |  |
| 5 or more times | 540 | 33.50 |  |  | 451 | 32.82 |  |  |
| *Fully mature group are the group who experienced the earliest pubertal timing | | | | | | | | |

| **Supplementary table 2:** Pairwise correlations between the four DNAm age acceleration markers (n=1,376) | | | | |
| --- | --- | --- | --- | --- |
|  | AgeAccelHannum | AgeAccelHorvath | AgeAccelLevine | AgeAccelGrim |
| AgeAccelHannum | 1 |  |  |  |
| AgeAccelHannum | 0.40 | 1 |  |  |
| AgeAccelLevine | 0.49 | 0.42 | 1 |  |
| AgeAccelGrim | 0.25 | 0.13 | 0.41 | 1 |

| **Supplementary table 3**. DNAm Age Acceleration at 53 years and conditional growth based on LMS method | | | | | | | | | |
| --- | --- | --- | --- | --- | --- | --- | --- | --- | --- |
|  |  | AgeAccelHannum | | AgeAccelHorvath | | AgeAccelLevine | | AgeAccelGrim | |
|  | N | Coefficient (95% CI) | P value | Coefficient (95% CI) | P value | Coefficient (95% CI) | P value | Coefficient (95% CI) | P value |
| *Relative weight gain* |  |  |  |  |  |  |  |  |  |
| RWG between birth and 2* years | 1,127 | -0.09 (-0.34, 0.16) | 0.48 | -0.09 (-0.34, 0.15) | 0.44 | -0.05 (-0.39, 0.29) | 0.76 | -0.08 (-0.38, 0.22) | 0.62 |
| RWG between 2 and 4 years | 1,065 | 0.11 (-0.15, 0.37) | 0.41 | 0.19 (-0.06, 0.44) | 0.13 | -0.10 (-0.46, 0.25) | 0.56 | -0.16 (-0.47, 0.14) | 0.30 |
| RWG between 4 and 7 years | 1,168 | -0.18 (-0.42, 0.06) | 0.15 | -0.08 (-0.32, 0.15) | 0.50 | -0.04 (-0.36, 0.29) | 0.81 | 0.04 (-0.25, 0.33) | 0.79 |
| RWG between 7 and 15 years | 1,161 | 0.16 (-0.07, 0.40) | 0.17 | 0.01 (-0.22, 0.24) | 0.93 | 0.25 (-0.07, 0.58) | 0.12 | 0.51 (0.22, 0.80) | 0.001** |
| *Linear growth* |  |  |  |  |  |  |  |  |  |
| CLG between 2 and 4 years | 1,085 | -0.06 (-0.32, 0.19) | 0.64 | 0.07 (-0.18, 0.32) | 0.59 | -0.37 (-0.72, -0.03) | 0.03*** | -0.24 (-0.54, 0.06) | 0.12 |
| CLG between 4 and 7 years | 1,204 | 0.09 (-0.13, 0.32) | 0.42 | -0.07 (-0.30, 0.15) | 0.52 | 0.13 (-0.18, 0.44) | 0.42 | 0.02 (-0.26, 0.29) | 0.91 |
| CLG between 7 and 15 years | 1,174 | 0.13 (-0.11, 0.36) | 0.29 | 0.04 (-0.19, 0.27) | 0.74 | 0.05 (-0.27, 0.37) | 0.76 | 0.15 (-0.14, 0.44) | 0.30 |
| Adjusted for age in months at 53 years and sex.  RWG: Relative weight gain i.e., standardised residuals from regression of present weight z-score on previous weight and height z-scores and present height z-score  CLG: Conditional linear growth i.e., Standardised residuals from regression of present height z-score on previous height and weight z-scores  *LMS method not applied due to no information about exact age.  **p=0.04 after Bonferroni correction  ***p=1.0 after Bonferroni correction | | | | | | | | | |

| **Supplementary table 4**. DNAm Age Acceleration at 53 years and SITAR height-tempo | | | | | | | | | |
| --- | --- | --- | --- | --- | --- | --- | --- | --- | --- |
|  |  | AgeAccelHannum | | AgeAccelHorvath | | AgeAccelLevine | | AgeAccelGrim | |
|  | N | Coefficient (95% CI) | P value | Coefficient (95% CI) | P value | Coefficient (95% CI) | P value | Coefficient (95% CI) | P value |
| *Women* |  |  |  |  |  |  |  |  |  |
| Height tempo (years) | 720 | 0.01 (-0.03, 0.06) | 0.49 | -0.02 (-0.06, 0.02) | 0.35 | -0.02 (-0.08, 0.03) | 0.42 | 0.02 (-0.02, 0.07) | 0.33 |
|  |  |  |  |  |  |  |  |  |  |
| *Men* |  |  |  |  |  |  |  |  |  |
| Height tempo (years) | 656 | -0.050 (-0.101, 0.002) | 0.06 | -0.02 (-0.07, 0.03) | 0.38 | -0.04 (-0.10, 0.03) | 0.30 | -0.001 (-0.063, 0.061) | 0.97 |
| Adjusted for age at interview (in months) at 53 years | | | | | | | | | |

| **Supplementary table 5.** DNAm Age Acceleration at 53 years and conditional growth adjusted for estimated cell composition | | | | | | | | | |
| --- | --- | --- | --- | --- | --- | --- | --- | --- | --- |
|  |  | AgeAccelHannum | | AgeAccelHorvath | | AgeAccelLevine | | AgeAccelGrim | |
|  | N | Coefficient (95% CI) | P value | Coefficient (95% CI) | P value | Coefficient (95% CI) | P value | Coefficient (95% CI) | P value |
| *Relative weight gain* |  |  |  |  |  |  |  |  |  |
| RWG between birth and 2 years | 1,127 | -0.12 (-0.34, 0.10) | 0.29 | -0.09 (-0.33, 0.14) | 0.44 | -0.11 (-0.40, 0.18) | 0.47 | -0.12 (-0.39, 0.16) | 0.40 |
| RWG between 2 and 4 years | 1,065 | 0.12 (-0.11, 0.34) | 0.31 | 0.21 (-0.04, 0.45) | 0.10 | -0.01 (-0.31, 0.29) | 0.95 | -0.07 (-0.35, 0.22) | 0.65 |
| RWG between 4 and 7 years | 1,168 | -0.10 (-0.32, 0.13) | 0.37 | -0.08 (-0.33, 0.16) | 0.50 | 0.14 (-0.16, 0.43) | 0.36 | 0.11 (-0.18, 0.39) | 0.46 |
| RWG between 7 and 15 years | 1,161 | 0.15 (-0.06, 0.36) | 0.16 | 0.02 (-0.22, 0.25) | 0.88 | 0.21 (-0.08, 0.49) | 0.16 | 0.40 (0.13, 0.68) | <0.001 |
| *Linear growth* |  |  |  |  |  |  |  |  |  |
| CLG between 2 and 4 years | 1,085 | 0.09 (-0.13, 0.31) | 0.44 | 0.15 (-0.10, 0.39) | 0.24 | -0.20 (-0.50, 0.10) | 0.18 | -0.18 (-0.46, 0.10) | 0.21 |
| CLG between 4 and 7 years | 1,204 | 0.03 (-0.17, 0.23) | 0.77 | -0.03 (-0.25, 0.19) | 0.78 | 0.07 (-0.19, 0.34) | 0.60 | -0.04 (-0.29, 0.22) | 0.77 |
| CLG between 7 and 15 years | 1,174 | 0.04 (-0.16, 0.25) | 0.67 | -0.01 (-0.24, 0.21) | 0.90 | -0.07 (-0.34, 0.21) | 0.64 | 0.16 (-0.11, 0.43) | 0.24 |
| Adjusted for age in months at 53 years and sex.  RWG: Relative weight gain i.e. standardised residuals from regression of present weight z-score on previous weight and height z-scores and present height z-scores  CLG: Conditional linear growth i.e. Standardised residuals from regression of present height z-score on previous height and weight z-scores | | | | | | | | | |

| **Supplementary table 6.** DNAm Age Acceleration at 53 years and pubertal timing adjusted for estimated cell composition | | | | | | | | | |
| --- | --- | --- | --- | --- | --- | --- | --- | --- | --- |
|  |  | AgeAccelHannum | | AgeAccelHorvath | | AgeAccelLevine | | AgeAccelGrim | |
|  | N | Coefficient (95% CI) | P value | Coefficient (95% CI) | P value | Coefficient (95% CI) | P value | Coefficient (95% CI) | P value |
| *Women* |  |  |  |  |  |  |  |  |  |
| Age at menarche (years) | 617 | -0.01 (-0.23, 0.20) | 0.92 | 0.003 (-0.240, 0.245) | 0.98 | -0.23 (-0.53, 0.07) | 0.13 | 0.07 (-0.22, 0.35) | 0.66 |
| Age at menarche |  |  |  |  |  |  |  |  |  |
| <12 years | 96 | Ref. |  | Ref. |  | Ref. |  | Ref. |  |
| ≥12 years | 521 | 0.02 (-0.73, 0.76) | 0.97 | 0.16 (-0.68, 1.00) | 0.70 | -0.04 (-1.09, 1.00) | 0.94 | 0.53 (-0.46,1.53) | 0.29 |
|  |  |  |  |  |  |  |  |  |  |
| *Men: Pubertal status at 14-15 years* | | |  |  |  |  |  |  |  |
| Fully mature* | 172 | Ref. |  | Ref. |  | Ref. |  | Ref. |  |
| Advanced puberty | 211 | 0.14 (-0.62, 0.89) | 0.27** | -0.40 (-1.22, 0.42) | 0.80** | 0.93 (-0.05, 1.90) | 0.05** | -0.31 (-1.26, 0.64) | 0.78** |
| Early puberty | 200 | 0.62 (-0.14, 1.39) |  | -0.13 (-0.97, 0.70) |  | 0.82 (-0.17, 1.81) |  | 0.15 (-0.82, 1.12) |  |
| Pre-pubertal | 73 | -0.18 (-1.21, 0.84) |  | -0.28 (-1.40, 0.84) |  | -0.52 (-1.84, 0.81) |  | -0.18 (-1.48, 1.11) |  |
|  |  |  |  |  |  |  |  |  |  |
| Fully mature | 172 | Ref. |  | Ref. |  | Ref. |  | Ref. |  |
| Later puberty | 484 | 0.29 (-0.36, 0.94) | 0.38 | -0.27 (-0.98, 0.44) | 0.45 | 0.67 (-0.18, 1.51) | 0.12 | -0.10 (-0.92, 0.72) | 0.81 |
| Adjusted for age in months at 53 years. *Fully mature group are the group who experienced the earliest pubertal timing. **p-value from lrtest comparing models with and without categorical puberty variable | | | | | | | | | |

| **Supplementary table 7.** DNAm Age Acceleration at 60-64 years and conditional growth | | | | | | | | | |
| --- | --- | --- | --- | --- | --- | --- | --- | --- | --- |
|  |  | AgeAccelHannum | | AgeAccelHorvath | | AgeAccelLevine | | AgeAccelGrim | |
|  | N | Coefficient (95% CI) | P value | Coefficient (95% CI) | P value | Coefficient (95% CI) | P value | Coefficient (95% CI) | P value |
| *Relative weight gain* |  |  |  |  |  |  |  |  |  |
| RWG between birth and 2 years | 402 | -0.11 (-0.55, 0.33) | 0.61 | -0.11 (-0.58, 0.36) | 0.64 | -0.21 (-0.83, 0.41) | 0.51 | -0.50 (-0.95, -0.05) | 0.03 |
| RWG between 2 and 4 years | 385 | 0.51 (0.08, 0.95) | 0.02 | 0.59 (0.13, 1.06) | 0.01 | 0.54 (-0.07, 0.40) | 0.08 | 0.05 (-0.41, 0.50) | 0.84 |
| RWG between 4 and 7 years | 441 | -0.39 (-0.80, 0.03) | 0.07 | -0.12 (-0.57, 0.33) | 0.61 | -0.20 (-0.79, 0.40) | 0.52 | -0.35 (-0.79, 0.10) | 0.13 |
| RWG between 7 and 15 years | 450 | 0.17 (-0.19, 0.54) | 0.35 | 0.03 (-0.39, 0.45) | 0.89 | 0.69 (0.12, 1.26) | 0.02 | 0.38 (-0.04, 0.79) | 0.08 |
|  |  |  |  |  |  |  |  |  |  |
| *Linear growth* |  |  |  |  |  |  |  |  |  |
| CLG between 2 and 4 years | 393 | -0.16 (-0.59, 0.26) | 0.45 | 0.21 (-0.25, 0.66) | 0.37 | -0.37 (-0.97, 0.22) | 0.22 | -0.10 (-0.54, 0.34) | 0.66 |
| CLG between 4 and 7 years | 441 | -0.11 (-0.48, 0.26) | 0.55 | -0.27 (-0.66, 0.13) | 0.19 | -0.17 (-0.69, 0.36) | 0.54 | -0.07 (-0.47, 0.32) | 0.71 |
| CLG between 7 and 15 years | 454 | 0.14 (-0.23, 0.50) | 0.46 | 0.51 (0.09, 0.93) | 0.02 | 0.72 (0.16, 1.29) | 0.01 | 0.26 (-0.16, 0.67) | 0.23 |
| Adjusted for age in months at 60-64 years and sex.  RWG: Relative weight gain i.e. standardised residuals from regression of present weight z-score on previous weight and height z-scores and present height z-score  CLG: Conditional linear growth i.e. Standardised residuals from regression of present height z-score on previous height and weight z-scores | | | | | | | | | |

| **Supplementary table 8.** DNAm Age Acceleration at 60-64 years and pubertal timing | | | | | | | | | |
| --- | --- | --- | --- | --- | --- | --- | --- | --- | --- |
|  |  | AgeAccelHannum | | AgeAccelHorvath | | AgeAccelLevine | | AgeAccelGrim | |
|  | N | Coefficient (95% CI) | P value | Coefficient (95% CI) | P value | Coefficient (95% CI) | P value | Coefficient (95% CI) | P value |
| *Women* |  |  |  |  |  |  |  |  |  |
| Age at menarche (years) | 212 | 0.26 (-0.14, 0.66) | 0.19 | 0.55 (0.13, 0.97) | 0.01 | 0.653 (0.002, 1.305) | 0.049 | 0.38 (-0.09, 0.85) | 0.11 |
| Age at menarche |  |  |  |  |  |  |  |  |  |
| <12 years | 34 |  |  |  |  |  |  |  |  |
| ≥12 years | 178 | 1.34 (-0.02, 2.70) | 0.05 | 1.09 (-0.36, 2.55) | 0.14 | 2.13 (-0.09, 4.36) | 0.06 | 1.50 (-0.09,3.10) | 0.06 |
|  |  |  |  |  |  |  |  |  |  |
| *Men: Pubertal status at 14-15 years* | | |  |  |  |  |  |  |  |
| Fully mature* | 73 | Ref. |  | Ref. |  | Ref. |  | Ref. |  |
| Advanced puberty | 92 | -0.70 (-2.11, 0.71) | 0.07** | -0.44 (-1.96, 1.08) | 0.28** | -0.70 (-2.54, 1.14) | 0.62** | -1.29 (-2.68, 0.09) | 0.10** |
| Early puberty | 81 | -0.41 (-1.86, 1.04) |  | -1.06 (-2.62, 0.50) |  | -1.65 (-3.54, 0.25) |  | -1.23 (-2.66, 0.20) |  |
| Pre-pubertal | 24 | -1.76 (-3.88, 0.35) |  | -1.80 (-4.08, 0.49) |  | -1.85 (-4.62, 0.92) |  | -1.29 (-3.38, 0.79) |  |
|  |  |  |  |  |  |  |  |  |  |
| Fully mature | 73 | Ref. |  | Ref. |  | Ref. |  | Ref. |  |
| Later puberty | 197 | -0.72 (-1.95, 0.51) | 0.25 | -0.86 (-2.19, 0.46) | 0.20 | -1.23 (-2.84, 0.37) | 0.13 | -1.27 (-2.47, -0.06) | 0.04 |
| Adjusted for age in months at 60-64 years. *Fully mature group are the group who experienced the earliest pubertal timing. **p-value from lrtest comparing models with and without categorical puberty variable | | | | | | | | | |
